# Global Path Planning Methods Based on the Relationship Between Traversability Capability and Terrain Matching

**DOI:** 10.3390/s26051472

**Published:** 2026-02-26

**Authors:** Zengbin Wu, Hongchao Zhang, Zhen Zhang, Da Jiang, Shuhui Li, Yunlong Sun

**Affiliations:** 1 School of Mechanical Science and Engineering, Huazhong University of Science and Technology, Wuhan 430074, China; 2 Northern Vehicle Research Institute, Beijing 100072, China

**Keywords:** terrain traversability, path planning, road network expansion, unstructured environment

## Abstract

In contrast to structured urban settings, road networks in post-disaster or unstructured wildland environments are often incomplete or compromised. Navigation in these contexts requires navigating complex terrains and mitigating potential hazards that impede unmanned ground vehicles (UGVs). While high-mobility off-road vehicles are specifically designed to traverse challenging features like ditches and steep slopes, traditional path planning algorithms often fail to exploit these capabilities. These algorithms typically suffer from a binary focus, either relying strictly on road networks or ignoring them altogether, thereby neglecting the synergy between infrastructure and vehicle mobility. This chapter introduces a global path planning method based on traversability analysis and terrain matching to bridge this gap. The methodology incorporates a grid-based traversability evaluation, a road network expansion algorithm for densifying critical segments, and a unified planning strategy. By correlating terrain characteristics with vehicle mobility limits and optimizing the road network density, the proposed framework achieves an integrated on-road and off-road planning solution that maximizes the operational efficiency of high-mobility vehicles in degraded environments.

## 1. Introduction

Path planning, a pivotal research area in intelligent systems, has received extensive attention and demonstrated broad application potential across aerial, terrestrial, and underwater domains [1,2]. In complex terrestrial environments, global path planning for autonomous ground vehicles (UGVs) aims to generate safe, efficient, and kinematically feasible trajectories. This process necessitates a systematic integration of multidimensional factors, including unstructured environmental constraints, vehicle kinematic limits, and safety regulations. To solve collision-free path search problems from initial to goal configurations, prevailing methodologies primarily leverage graph search-based and sampling-based algorithms.

Regarding traversability analysis in complex environments, field operations must account for the inherent challenges of unstructured terrain. Off-road environments, such as mountainous regions, wetlands, and deserts, lack well-defined boundaries and artificial landmarks. Consequently, traversability assessment in these areas encounters significant difficulties due to irregular topography, environmental stochasticity, and nonlinear vehicle–terrain interaction. Traditional assessment methods often rely on rigid geometric thresholds—such as defining traversability based solely on a maximum slope of 30°. However, such deterministic approaches struggle to accommodate the requirements of multimodal data fusion and dynamic operational conditions [3,4].

Current research in terrain traversability is transitioning from single-feature heuristics toward comprehensive evaluation frameworks that integrate multi-source data and multidimensional metrics [5]. Researchers have significantly enhanced the precision and reliability of assessment outcomes by optimizing algorithmic models and employing machine learning and deep learning techniques to quantitatively analyze key factors, including terrain gradient, surface roughness, and obstacle distribution [6]. Concurrently, the research paradigm has shifted from static assessment to real-time dynamic evaluation. From a technical perspective, the widespread adoption of high-resolution remote sensing, 3D LiDAR scanning, and multispectral sensors has provided robust tools for high-fidelity terrain data acquisition [7]. Furthermore, advancements in edge and cloud computing have facilitated the efficient processing and real-time analysis of massive datasets, further advancing the state-of-the-art in terrain traversability assessment [8].

Driven by the rapid advancement of robotics, autonomous driving, and unmanned systems, terrain traversability assessment (TTA) methodologies are continuously evolving to address increasingly diverse operational requirements. Current state-of-the-art approaches are broadly categorized into geometry-based, appearance-based, hybrid, learning-based, and physics-driven methods, each offering distinct perspectives for robust traversability analysis. Geometry-based methods are extensively employed when explicit terrain elevation data is available. This paradigm evaluates traversability by correlating topographic features with a vehicle’s mechanical constraints, such as its maximum approach/departure angles and ground clearance [3]. Prevailing techniques include grid-based discretization, which partitions the environment into cells to compute local morphological properties [9], and statistical modeling, which utilizes Gaussian distributions or kernel functions (e.g., Radial Basis Functions) to characterize terrain irregularities [10]. While these methods provide high computational efficiency and clear physical interpretability—making them suitable for real-time navigation in structured settings—they rely heavily on high-fidelity geometric data. Consequently, they often exhibit limited sensitivity to non-geometric hazards, such as yielding soil or compressible vegetation, which poses significant challenges in complex natural environments [11].

Appearance-based methods primarily utilize RGB imagery or infrared data to delineate navigable regions based on visual cues such as color and texture [12]. Classical computer vision approaches leverage HSV color space analysis and Local Binary Patterns (LBP) for terrain classification [13]. Conversely, modern deep learning architectures employ Convolutional Neural Networks (CNNs), such as ResNet, for high-level feature extraction and semantic segmentation of terrain images [14]. These methods are less dependent on precise geometric information and excel in texture-rich environments like forests and deserts. Nevertheless, their performance is highly contingent on ambient conditions; environmental fluctuations, such as varying illumination and shadows, can degrade classification reliability and compromise overall assessment accuracy [15].

To mitigate the inherent limitations of unimodal approaches, hybrid frameworks have emerged to fuse disparate data sources. These methods integrate geometric and appearance-based information—such as LiDAR point clouds combined with RGB imagery—or incorporate multi-modal sensor data including inertial and tactile feedback to enhance robustness [7]. Representative architectures include semantic-geometric fusion, where LiDAR-derived elevation maps are augmented by semantic labels from RGB segmentation to generate comprehensive cost maps [16]. Additionally, multimodal learning paradigms utilize deep neural networks to extract joint representations from heterogeneous data [17]. While hybrid methods effectively synthesize multidimensional information to navigate complex terrains like muddy slopes, the underlying fusion algorithms are computationally intensive and demand substantial onboard processing resources [18].

Machine learning and deep learning (ML/DL) paradigms have demonstrated significant potential in terrain traversability assessment. Historically, classical ML models such as Support Vector Machines (SVM) and Random Forests were predominantly employed for discrete classification tasks [19]. With the advent of deep learning, CNN-based processing of elevation maps has achieved classification accuracies surpassing 90%, while Transformer architectures have further enhanced the modeling of long-range spatial dependencies [20]. Beyond simple binary classification, contemporary research has transitioned toward multi-objective regression, enabling the continuous prediction of performance metrics such as attainable speed, energy consumption, and acceleration. These advancements transcend the limitations of traditional discrete labels. Furthermore, sim-to-real transfer techniques, bolstered by domain adaptation, allow models trained on synthetic datasets to be effectively deployed in real-world environments, thereby broadening the operational scope of autonomous systems [21].

Physics-driven methods are grounded in terramechanics and vehicle dynamics, evaluating traversability by formulating coupled mechanical-kinematic equations [22]. For instance, the cone index (CI) method quantifies the impact of soil strength on tracked vehicle mobility and generates traversal costs by incorporating terrain gradients—a methodology successfully applied in complex terrain studies, such as those involving the Tibetan Plateau [23]. Additionally, multibody dynamics (MBD) frameworks leverage simulation environments like ADAMS or RecurDyn to model high-fidelity vehicle–terrain interactions. These simulations generate precise physical data that serve as a rigorous foundation for mobility assessment [24]. Despite their accuracy, physics-based approaches are often characterized by high modeling complexity and the inherent difficulty of acquiring precise physical parameters in situ, which can constrain their real-time applicability in highly dynamic or unstructured scenarios [25,26].

Traversability analysis in battlefield environments must account for both geographical terrain characteristics and hostile threat factors; however, publicly accessible information regarding the latter remains notably limited. In scenarios with deterministic map data, graph-search methodologies—specifically the traditional Dijkstra algorithm and its priority-queue-optimized variants—are common selections. The standard Dijkstra algorithm, however, suffers from high time complexity due to its exhaustive traversal of unvisited vertices. In large-scale path planning, this computational inefficiency becomes increasingly problematic, resulting in excessive latency that fails to meet the real-time requirements of dynamic environments [3]. While the A* algorithm enhances search efficiency by incorporating a heuristic function, accurately designing an appropriate heuristic for complex terrains is non-trivial. It demands profound environmental insights and domain expertise, which increases the difficulty and uncertainty of algorithmic implementation [6].

For complex environments that are difficult to model precisely, sampling-based algorithms, such as the Rapidly-exploring Random Tree (RRT), have become prevalent. However, due to their inherent stochasticity, these methods often yield inconsistent trajectories characterized by low search efficiency and variable path optimality. In safety-critical scenarios requiring high-quality paths—such as disaster response and emergency rescue—standard RRT variants may fail to satisfy operational constraints. Consequently, additional path-smoothing and optimization steps are typically required, which inevitably increases the overall algorithmic complexity and computational load [7]. To address multi-constraint challenges across diverse domains, Reinforcement Learning (RL) has gained prominence for its robust adaptability in uncertain settings [8]. Nevertheless, RL-based approaches are often hindered by data-intensive training processes, slow convergence, and a susceptibility to local optima, presenting significant hurdles for their practical deployment in real-world tasks.

The core architecture of graph-search path planning primarily comprises graph construction, node expansion strategies, and path evaluation/optimization. These components provide the necessary framework for achieving efficient and precise global navigation. Specifically, graph construction and expansion strategies can be synthesized into a unified design to address planning problems in distinct environmental contexts. Based on their application, existing algorithms are generally categorized into two paradigms: those optimized for structured road networks and those tailored for unstructured off-road environments. Path planning algorithms tailored for road networks are predominantly applied in urban transportation and logistics distribution. To enhance the performance and practicality of these algorithms within complex urban infrastructures, researchers have explored innovative graph construction methodologies. A notable approach involves generating dynamic graphs by integrating Geographic Information System (GIS) data with real-time traffic streams [9]. In this framework, static topology and node locations are derived from GIS, while edge weights are dynamically updated to reflect current traffic conditions—such as adjusting travel costs based on congestion levels—ensuring the graph realistically mirrors the network state. Regarding the attribute definition of nodes and edges, recent studies have moved beyond conventional metrics like distance and travel time to incorporate multidimensional constraints, including speed limits, lane configurations, and traffic regulations [10]. By annotating graph components with these attributes, algorithms can comprehensively account for operational constraints, ensuring the legality and feasibility of the planned paths.

To address the challenges of graph construction in unstructured field environments, various methodologies have been proposed to handle the lack of predefined topologies. Recognizing the critical influence of topography on traversability, several studies leverage Digital Elevation Model (DEM) data to generate terrain-aware graph structures [11]. By processing DEM data, the environment is discretized into distinct regions, each functioning as a node within the graph. Connectivity between nodes is determined by physical factors such as slope gradients and surface roughness. For regions with gentle slopes and uniform terrain, connections are established with low edge weights to reflect minimal traversal costs. Conversely, for high-risk areas—such as steep cliffs and ravines—connections are either pruned or assigned prohibitive edge weights to ensure avoidance. To enhance obstacle representation fidelity, alternative approaches integrate multi-modal perception from LiDAR and visual sensors to detect and model obstacles precisely [12]. This occupancy information is embedded into the graph; specifically, connectivity in obstacle-dense zones is restricted, or traversal costs are artificially inflated to guide the algorithm toward safer trajectories.

Regarding grid discretization, researchers have introduced multi-resolution and adaptive grid techniques to mitigate the issues of excessive graph size and high computational overhead inherent in uniform partitioning [13]. Multi-resolution strategies apply variable grid granularity based on environmental complexity: fine-grained grids are deployed in areas with abrupt terrain changes or dense obstacles to enhance precision, while coarse grids are utilized in open, flat terrain to improve computational efficiency. Similarly, adaptive grid partitioning dynamically adjusts grid density based on feedback during the search process, aligning the discretization resolution with the actual requirements of the path search and thereby optimizing overall algorithmic performance.

To address the challenges posed by rugged and complex terrain, researchers have introduced various enhancements to traditional graph-search algorithms. Hierarchical graph-based methods partition the environment based on elevation profiles and obstacle distributions, establishing inter-layer transitions that allow the algorithm to navigate across multi-layered architectures. This approach enhances search flexibility and helps the algorithm circumvent local optima. For instance, in mountainous search-and-rescue (SAR) operations, terrain can be categorized into semantic strata—such as the base, slopes, and summits—with distinct graph structures for each level. Transitions between these layers enable the search to resume even if it encounters a local optimum within a single stratum, ensuring the identification of a globally optimal path [14]. Furthermore, multi-scale search algorithms employ a coarse-to-fine strategy: an initial rapid search on a large-scale graph determines a global trajectory corridor, followed by a refined search on small-scale local graphs. This significantly improves both computational throughput and planning precision. To handle dynamic environments, modern algorithms incorporate real-time monitoring and online replanning mechanisms [15]. Onboard sensors facilitate instantaneous data acquisition, triggering replanning upon detecting dynamic obstacles. Incremental search techniques are often employed to optimize resource consumption by restricting updates to local regions affected by environmental changes rather than recomputing the entire global path [7]. Additionally, path-smoothing algorithms eliminate sharp turns to satisfy the vehicle’s kinematic constraints, ensuring the safe operation of rescue platforms in complex settings.

Despite these global advancements, existing methodologies remain insufficient for the urgent requirements of specialized rescue vehicles in post-disaster scenarios where road networks are compromised or severed. While current research incorporates natural terrain and infrastructure [16], the integrated assessment of rapid-onset dynamic hazards—such as secondary structural collapses, landslides, and signal degradation—alongside terrain traversability remains neither timely nor sufficiently accurate. This inability to identify high-risk zones in real-time jeopardizes the safety of rescue platforms. Furthermore, regarding vehicle performance, although the impact of varying kinematic capabilities is recognized [17], there is a lack of systematic integration of the unique off-road characteristics of specialized vehicles under extreme conditions. Specifically, factors such as soil-bearing capacity, geometric clearance, and maneuverability limits in hazardous environments are frequently overlooked. This deficiency prevents the precise quantification of vehicle traversal limits, thereby compromising the overall accuracy of global path planning. The main contributions of this work are summarized as follows:

(i) Fine-grained traversability evaluation based on vehicle–terrain matching:
  Addressing the inability of conventional algorithms to distinguish between structured roads and unstructured terrain, we propose a high-fidelity traversability evaluation framework. By discretizing the environment into grid cells and mapping topographic features against vehicle-specific mobility metrics—such as trench-crossing and climbing capacities—this method provides a quantitative assessment of passability, enabling autonomous platforms to fully leverage their inherent off-road capabilities.
(ii) Hybrid global path planning for compromised infrastructure: To navigate the fragmented or incomplete road networks typical of disaster zones, we develop a hybrid global path planning algorithm that synergistically integrates road-network expansion with unstructured off-road planning. This methodology densifies the road network in critical segments to optimize travel efficiency while facilitating seamless transitions to off-road modes when infrastructure is severed, effectively unifying structured and unstructured navigation.
(iii) Theoretical stability and convergence guarantees: A rigorous theoretical analysis is established to guarantee the stability of the learning components within the planning system. We provide a convergence theorem for the neural network utilized in traversability cost estimation, proving that the updated parameters—including weights and structural coefficients—converge to an optimal and consistent solution. This mathematical foundation ensures the reliability of navigation decisions within complex optimization landscapes.
(iv) Empirical validation in complex rescue scenarios: Extensive simulation experiments are conducted to validate the proposed framework. The results demonstrate that our approach significantly outperforms traditional road-centric or pure off-road algorithms in terms of path feasibility and transit efficiency. Specifically, the method exhibits superior adaptability in extreme rescue environments characterized by steep gradients, deep fissures, and severely degraded road networks.

The rest of the paper is organized as follows. In Section 2, we describe the accessibility evaluation method based on hierarchical overlay. Section 3 details the multi-layer fusion framework for passability evaluation. We provide the proof of neural network convergence in Section 4. Section 5 presents the numerical experiments and comparative analysis. Finally, conclusions and future works are discussed in Section 6.

## 2. Accessibility Evaluation Method Based on Hierarchical Overlay

As mentioned previously, numerous factors affect unmanned vehicle traversability on land battlefields. The area requiring traversal is divided into a grid-based format, and map attribute feature layers are constructed based on evaluation factors. Each layer is generated according to a specific evaluation factor, and finally an overlay algorithm is employed to fuse multiple layers into a traversability characteristic map to support subsequent path planning.

In the single-factor traversability coefficient analysis, by defining the traversability coefficient K∈[0,1] (where larger *K* values indicate better traversability), natural wilderness environmental factor values are mapped into three categories: easily passable, passable, and impassable. Prior to the derivation of these formulas, all raw data underwent a comprehensive pre-processing stage, including normalization, to ensure consistency across varying environmental conditions. The value ranges for these three categories are represented in Table 1. The analytical expressions for traversability presented in Table 2, Table 3, Table 4, Table 5, Table 6, Table 7, Table 8, Table 9, Table 10, Table 11, Table 12, Table 13 and Table 14 were established through a combination of inherent vehicle performance specifications and empirical data collected from multiple rounds of rigorous testing.

(1) Impact of Altitude on Unmanned Vehicle Traversability

Altitude directly affects engine power output. Higher altitudes result in thinner air, which can lead to incomplete fuel combustion, reduced engine power, and decreased mobility performance of vehicle equipment. The vehicles be designed to operate continuously at altitudes below 3000 m and requires wheeled vehicles to generally possess the capability to operate normally at altitudes up to 4700 m. Based on practical engineering considerations, an altitude of 3000 m represents the mandatory requirement for tracked vehicles, while an altitude of 4700 m represents the mandatory requirement for wheeled vehicles.

The relationships between traversability coefficients and altitude for tracked vehicles and wheeled vehicles are constructed as shown in Table 2 and Table 3, respectively.

**Table 2:** Mapping Relationship between Altitude and Traversability Coefficient for Tracked Vehicles.

Value *X* (m)	Traversability Coefficient	Mapping Relationship
[0,1000)	0.8≤K≤1	$K=-\frac{1}{5000}X+1$
[1000,3000)	0.4≤K<0.8	$K=-\frac{1}{5000}X+1$
[3000,5000)	0≤K<0.4	$K=-\frac{1}{5000}X+1$
[5000,+∞)	0	K=0

From Table 2, we define a monotonically decreasing linear relationship between the input factor *X* (e.g., altitude) and the traversability coefficient *K*. This negative correlation is consistent with empirical physical laws; for instance, as altitude increases, the engine performance or environmental safety typically degrades, leading to a reduction in vehicle throughput capacity. For the specific case of altitude in Table 2, the mapping relationship is defined as:

(1) $$K=\left\{\begin{array}{cc} -\frac{1}{5000}X+1, & 0\leq X<5000\\ 0, & X\geq 5000 \end{array}\right.$$

This approach represents a preliminary macro-scale exploration of traversability. Currently, we employ the control variable method, where each coefficient *K* is calculated based on its correlation with a single dominant factor to maintain computational efficiency and clarity in grade classification.

**Table 3:** Mapping Relationship between Altitude and Traversability Coefficient for Wheeled Vehicles.

Value *X* (m)	Traversability Coefficient	Mapping Relationship
[0,2000)	0.8≤K≤1	$K=-\frac{1}{10000}X+1$
[2000,4700)	0.4≤K<0.8	$K=-\frac{1}{6750}X+\frac{148}{135}$
[4700,6000)	0≤K<0.4	$K=-\frac{1}{3250}X+\frac{24}{13}$
[6000,+∞)	0	K=0

(2) Impact of Terrain on Unmanned Vehicle Traversability

Terrain is decomposed into several aspects: step terrain, trench terrain, and gentle slope terrain, where gentle slopes are further divided into longitudinal slopes and lateral slopes. These terrain types typically do not exist simultaneously. The off-road vehicles usually need overcome vertical wall obstacles of no less than 0.8 m, while other tracked vehicles should overcome obstacles of no less than 0.6 m. The mapping relationship between height difference and traversability coefficient for tracked equipment is established as shown in the following Table 4:

**Table 4:** Mapping Relationship between Step Height Difference and Traversability Coefficient for Tracked Vehicles.

Value *X* (m)	Traversability Coefficient	Mapping Relationship
[0,0.3)	0.8≤K≤1	$K=-\frac{2}{3}X+1$
[0.3,0.8)	0.4≤K<0.8	$K=-\frac{4}{5}X+\frac{26}{25}$
[0.8,1.5)	0≤K<0.4	$K=-\frac{4}{7}X+\frac{6}{7}$
[1.5,+∞)	0	K=0

The wheeled vehicles generally overcome vertical obstacles of no less than 0.4 m. The mapping relationship between height difference and traversability coefficient for wheeled equipment is established as shown in the following Table 5:

**Table 5:** Mapping Relationship between Step Height Difference and Traversability Coefficient for Wheeled Vehicles.

Value *X* (m)	Traversability Coefficient	Mapping Relationship
[0,0.2)	0.8≤K≤1	K=−X+1
[0.2,0.4)	0.4≤K<0.8	$K=-2X+\frac{6}{5}$
[0.4,1.5)	0≤K<0.4	$K=-\frac{4}{11}X+\frac{6}{11}$
[1.5,+∞)	0	K=0

It is required that wheeled vehicles generally overcome trenches with widths of no less than 0.8 m. For 6 × 6 equal-wheelbase vehicles, the trench crossing width should generally be no less than 1.2 m, while for 8 × 8 vehicles it should be no less than 1.8 m. The mapping relationship between trench width and traversability coefficient for wheeled equipment is established as shown in the following Table 6:

**Table 6:** Mapping Relationship between Trench Crossing Width and Traversability Coefficient for Wheeled Vehicles.

Value *X* (m)	Traversability Coefficient	Mapping Relationship
[0,0.4)	0.8≤K≤1	$K=-\frac{1}{2}X+1$
[0.4,0.8)	0.4≤K<0.8	$K=-X+\frac{6}{5}$
[0.8,1.8)	0≤K<0.4	$K=-\frac{2}{5}X+\frac{18}{25}$
[1.8,+∞)	0	K=0

The maximum climbing gradient for ground assault vehicles and combat service vehicles should be no less than 30°. The maximum climbing gradient for wheeled vehicles should generally be no less than 30°. The mapping relationship between gradient and traversability coefficient for equipment is established as shown in the following Table 7:

**Table 7:** Mapping Relationship between Gentle Slope Gradient and Traversability Coefficient.

Value *X*	Traversability Coefficient	Mapping Relationship
[0,15)	0.8≤K≤1	$K=-\frac{1}{75}X+1$
[15,30)	0.4≤K<0.8	$K=-\frac{2}{75}X+\frac{6}{5}$
[30,35)	0≤K<0.4	$K=-\frac{1}{150}X+\frac{3}{5}$
[35,90)	0	K=0

The maximum lateral slope gradient for wheeled vehicles should generally be no less than 17°. The mapping relationship between slope gradient and traversability coefficient for equipment is established as shown in the following Table 8:

**Table 8:** Mapping Relationship between Lateral Slope Gradient and Traversability Coefficient.

Value *X*	Traversability Coefficient	Mapping Relationship
[0,10)	0.8≤K≤1	$K=-\frac{1}{5}X+1$
[10,16)	0.4≤K<0.8	$K=-\frac{1}{15}X+\frac{22}{15}$
[16,20)	0≤K<0.4	$K=-\frac{1}{10}X+2$
[20,90)	0	K=0

Water bodies are decomposed into two aspects: water depth and water surface width. It is required that the fording depth of vehicles should generally be no less than 1.3 m. Therefore, the mapping relationship between water depth and traversability coefficient for equipment is established as shown in the following Table 9:

**Table 9:** Mapping Relationship between Water Body Depth and Traversability Coefficient.

Value *X* (m)	Traversability Coefficient	Mapping Relationship
[0,0.5)	0.8≤K≤1	$K=-\frac{2}{5}X+1$
[0.5,1.3)	0.4≤K<0.8	$K=-\frac{1}{2}X+\frac{21}{20}$
[1.3,2)	0≤K<0.4	$K=-\frac{4}{7}X+\frac{8}{7}$
[2,+∞)	0	K=0

There are no explicit regulations for the water surface width that vehicles can ford. Based on engineering practice, except for amphibious vehicles, during testing, the water surface width is comparable to the vehicle length. Increasing water surface width poses risks of failures during traversal. The mapping relationship between water surface width and traversability coefficient for equipment is established as shown in the following Table 10:

**Table 10:** Mapping Relationship between Water Width and Traversability Coefficient.

Value *X* (m)	Traversability Coefficient	Mapping Relationship
[0,1)	1	K=1
[1,3)	0.8≤K≤1	$K=-\frac{1}{10}X+\frac{11}{10}$
[3,5)	0.4≤K<0.8	$K=-\frac{1}{5}X+\frac{7}{5}$
[5,7)	0≤K<0.4	$K=-\frac{1}{5}X+\frac{7}{5}$
[7,+∞)	0	K=0

Typical surface covers include human-made cement roads and asphalt roads, as well as naturally formed grasslands, shrublands, forests, and exposed soil, where exposed soil significantly affects unmanned vehicle traversability depending on material composition and moisture content. Ice and snow are also included in the surface cover category. By default, human-made roads serving social life have a traversability coefficient of 0.9, grasslands have a traversability coefficient of 0.6, shrublands have traversability coefficients of 0.1 (wheeled) and 0.5 (tracked), and ice/snow surfaces have traversability coefficients of 0.4 (wheeled) and 0.6 (tracked). For forest traversability analysis, three aspects are mainly considered: tree spacing, diameter at breast height, and canopy closure. Having combat unmanned vehicles challenge trees is unwise; therefore, only the impact of tree spacing on traversability needs to be considered.

For dry hard road surfaces, the larger the soil particles, the worse the soil compressibility, and the more difficult it is for equipment to traverse. The mapping relationship between dry soil particle diameter and traversability coefficient for equipment is established as shown in the following Table 11:

**Table 11:** Mapping Relationship between Dry Surface Soil Particle Diameter and Traversability Coefficient.

Soil Type	Value *X* (mm)	Traversability Coefficient	Mapping Relationship
Gravel Soil	[2,10)	0<K≤0.5	$K=-\frac{1}{6}X+\frac{5}{6}$
Sandy Soil	[0.5,2)	0.5<K≤0.7	$K=-\frac{4}{39}X+\frac{55}{78}$
Loam	[0.02,0.5)	0.7<K≤0.9	$K=-\frac{25}{6}X+\frac{109}{120}$
Clay	(0,0.02)	0.9<K<1	K=−50X+1

For water-containing soil, the smaller the soil particles, the worse the soil permeability, the higher the degree of road muddiness, and the more difficult it is for equipment to traverse. The mapping relationship between water-containing soil particle size and traversability coefficient for equipment is established as shown in the following Table 12:

**Table 12:** Mapping Relationship between Water-Containing Surface Soil Particle Diameter and Traversability Coefficient.

Soil Type	Value *X* (mm)	Traversability Coefficient	Mapping Relationship
Gravel Soil	[2,10)	0.8<K≤1	$K=\frac{1}{15}X+\frac{2}{3}$
Sandy Soil	[0.5,2)	0.6<K≤0.8	$K=\frac{4}{39}X+\frac{116}{195}$
Loam	[0.02,0.5)	0.4<K≤0.6	$K=\frac{25}{6}X+\frac{47}{120}$
Clay	(0,0.02)	0<K<0.4	K=200X

For tracked vehicles, the vehicle width is generally no greater than 4 m, and the vehicle length is generally no greater than 10 m. Considering vehicles traversing through gaps between trees, the mapping relationship between tree spacing and traversability coefficient for tracked equipment is established as shown in the following Table 13:

**Table 13:** Mapping Relationship between Forest Tree Spacing and Traversability Coefficient for Tracked Vehicles.

Value *X* (m)	Traversability Coefficient	Mapping Relationship
[10,+∞)	1	K=1
[7,10)	0.8≤K<1	$K=\frac{1}{15}X+\frac{1}{3}$
[5,7)	0.4≤K<0.8	$K=\frac{1}{5}X-\frac{3}{5}$
[0,5)	0≤K<0.4	$K=\frac{2}{25}X$

For wheeled vehicles, the vehicle width is generally no greater than 3 m, and the vehicle length is generally no greater than 8 m. Considering vehicles traversing through gaps between trees, the mapping relationship between tree spacing and the traversability coefficient for wheeled equipment is established as shown in the following Table 14:

**Table 14:** Mapping Relationship between Forest Tree Spacing and Traversability Coefficient for Wheeled Vehicles.

Value *X* (m)	Traversability Coefficient	Mapping Relationship
[7,+∞)	1	K=1
[5,7)	0.8≤K<1	$K=\frac{1}{10}X+\frac{3}{10}$
[3,5)	0.4≤K<0.8	$K=\frac{1}{5}X-\frac{1}{5}$
[0,3)	0≤K<0.4	$K=\frac{2}{15}X$

**Figure 1:**
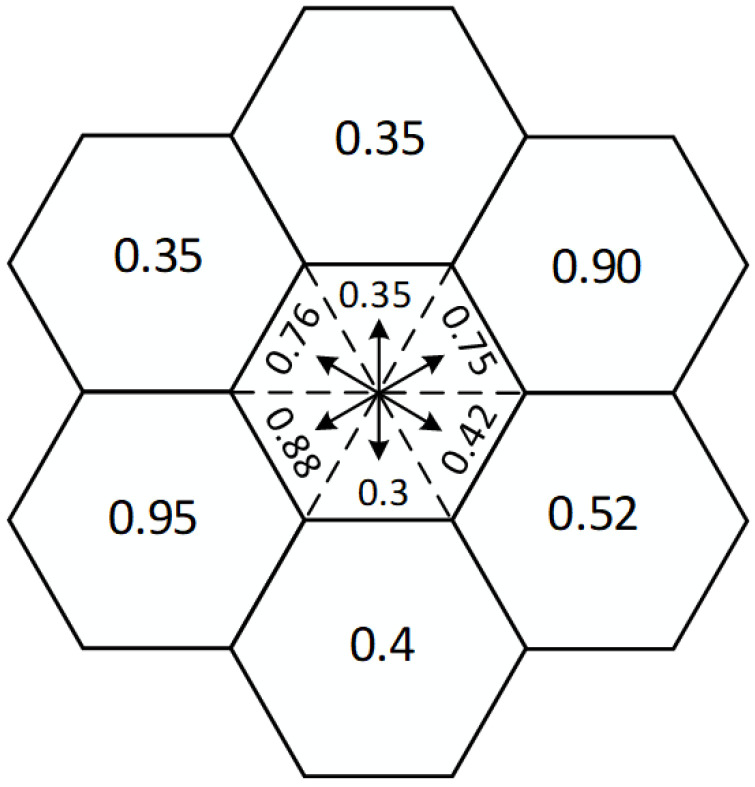
Basic schematic of the hexagonal grid with directional features.

As for the hexagonal grid-based layering overlay method. However, this method has some limitations, as it overlooks the directional impact of path orientation on accessibility. Factors such as longitudinal slope, side slope, vertical obstacles, and trenches analyzed earlier all have directional characteristics. Therefore, in this paper, the hexagonal grid is extended to incorporate directional features, as illustrated in Figure 1. The hexagonal grid is divided into 6 directions, and the obstacle characteristics formed by these 6 directions are coupled with the passability of the unmanned vehicle. This analysis results in an evaluation of passability for each direction. As a result, the hexagonal grid is extended into a directed graph, which facilitates accurate path planning.

## 3. Multi-Layer Fusion for Passability Evaluation

To evaluate the factors affecting passability, a quantitative assessment is performed on the hexagonal grid, resulting in layered evaluation values. The issue of how to perform multi-layer fusion is a key challenge. Traditional methods such as minimum value selection, averaging, or multiplication followed by remapping have limitations and cannot meet the complex needs of passability evaluation in wilderness environment conditions. Therefore, based on the principle of layering and by drawing inspiration from the Mixture of intelligent model, a neural network-based fitting method is proposed to learn from datasets obtained from simulation software, achieving multi-layer fusion.

During the preprocessing phase, individual evaluations are conducted as described earlier. Based on the results of these individual evaluations, classification is performed, and an inference model is established for each class. The inference model uses a BP (Backpropagation) neural network trained on the dataset. Compared with traditional multi-factor overlay analysis methods based on physical models, this model can cover all ground categories and factors, and it offers higher computational efficiency. As mentioned earlier, there are 16 factors that affect the passability of unmanned vehicles in the battlefield. However, these 16 factors are not independent; they still exhibit coupling relationships. For example, if water is present, the terrain will not be in an icy or dry state. Therefore, the coupling relationships among the 16 factors need to be studied and categorized.

Vertical obstacles, trenches, longitudinal slope, and side slope represent terrain features and are grouped into three categories, as shown in Table 15.

In the table, ‘1’ indicates that the corresponding factor does not exist, indirectly suggesting that the factor does not have an impact. The term ‘rand’ means that the parameter is variable and determined based on the local wilderness environment and the evaluation method described earlier, thus possessing randomness.

Water depth, surface width, structural road surfaces, grassland, shrubs, snow and ice, dry ground, and water-containing surfaces are eight factors that characterize surface features, and they exhibit coupling relationships. These factors are grouped into 10 categories, as shown in Table 16. In this table, ‘1’ indicates the absence of a corresponding factor, indirectly suggesting no impact of the factor. The term ‘rand’ represents a variable parameter, determined based on the local wilderness environment and the evaluation method described earlier, thus possessing randomness.

Additionally, altitude is independent and generated randomly. The vegetation spacing factor is categorized into two types: one with trees and one without. In the case with trees, the impact factor is calculated based on the tree spacing, while in the case without trees, the factor is set to 1, indicating no impact on passability. As a result, the wilderness environment is classified into 240 categories.

### 3.1. Support Vector Machine Classification Model

Support Vector Machine (SVM) is used to construct an optimal hyperplane to realize classification or regression tasks. Its main advantage lies in its ability to efficiently process high-dimensional feature spaces for classification tasks, especially in scenarios requiring high-dimensional classification, such as terrain, water, and wilderness environments. This makes it an ideal choice for classification tasks in complex wilderness environments.

For a continuous dataset ${\left\{\left(x_{i},y_{i}\right)\right\}}_{i=1}^{N}$, where $x_{i}\in R^{d}$ is the feature vector, and $y_{i}\in \left\{1,2,\ldots ,K\right\}$ is the class label, the model can be expressed by an error-correcting output code (ECOC) framework. In this framework, the SVM is used to solve multi-class classification tasks. At the same time, in constructing the dataset, it is important to evaluate the influence of differences in the class labels on the model’s ability, and use the class-specific feature vectors $x_{i}\in \left(0,1\right)$ to normalize the output of the communication evaluation results, ensuring the model’s standardization.

In SVM, the kernel function and the selection of its parameters are crucial for optimizing the model’s training process. Currently, the radial basis function (RBF) kernel is widely used. The parameters of the RBF kernel are highly sensitive to the data. If appropriate settings are not selected, the model may overfit or underperform. The Sigmoid kernel also satisfies the Mercer’s condition, indicating that it is a valid kernel function. Additionally, polynomial kernels, such as the Polynomial Kernel, can map features to higher-dimensional spaces, and the function is expressed as:

(2) $$K\left(x_{i},x_{j}\right)={\left(\gamma x_{i}^{T}x_{j}+c_{0}\right)}^{d},$$

where γ>0 is a kernel parameter, $c_{0}$ is a constant term, and *d* is the polynomial degree. The kernel parameters must be self-adjusted according to the dataset, with $\gamma =1/D_{\mathrm{median}}$, where $D_{\mathrm{median}}$ is the median distance between data points. This adjustment allows the kernel function to be adapted to the dataset and helps improve the model’s classification performance. Moreover, the influence of higher-dimensional spaces on the feature distribution can be better captured by non-linear kernel functions, enhancing the model’s ability to handle non-linear data.

**Figure 2:**
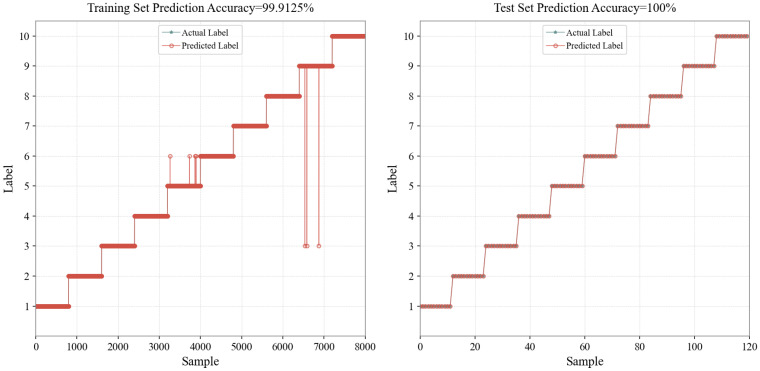
Prediction results based on the SVM model.

From the experimental results shown in Figure 2, it can be observed that the Support Vector Machine (SVM) model achieves a prediction accuracy of 99.9125% on the training set, and 100% accuracy on the testing set so that some lines overlapped. Specifically, the training set consists of 8000 data points, with 7 misclassified samples, indicating that the model has a strong fitting ability for the training data. When the trained model was used to predict the testing set, consisting of 120 independent samples collected from real wilderness environments, all the samples were correctly classified.

**Figure 3:**
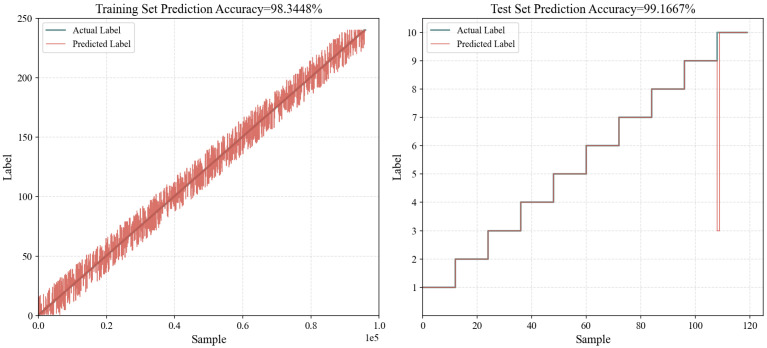
Robustness test results based on the SVM model.

To verify the robustness of the model in training with a large number of categories, a dataset similar to the one in Table 16 was created with 240 categories. For each category of wilderness environment, 400 data samples were generated to form the training set. To test the robustness of the model, the testing set used the same data. The results after training are shown in Figure 3. Any minor uncertainties in the classification stage are smoothed during the regression stage, preventing drastic fluctuations in the final path cost calculation. This integration ensures that the navigation system remains reliable even in complex transitional environments.

### 3.2. Backpropagation Neural Network

The Backpropagation Neural Network (BP Neural Network) is used here to approximate the nonlinear relationship between factors. The goal is to use the model to establish a predictive model for various wilderness environment factors. Specifically, including classes 24, 41, 52, 67, 79, and 188, were selected to build the model, validating the neural network’s generalization ability. Table 17 provides the initial data for the 24 category.

Since the optimization of each class’s feature variables differs, the input vector is $x=(x_{1},x_{2},\ldots ,x_{n})$, where each input point $x_{i}$ is in the range [0,1] for i=1,2,…,12. The BP neural network structure is used to model the nonlinearity, with a hidden layer having 10 neurons, and a second hidden layer containing 5 neurons. The neural network’s learning strategy is based on the backpropagation algorithm, using the purelin activation function for the output layer, and the sigmoid activation function for the hidden layers. The dataset was partitioned into training, validation, and testing sets with a distribution of 80%, 10%, and 10%, respectively. To ensure the robustness of the learning process and mitigate the impact of random data partitioning, a 10-fold cross-validation protocol was implemented. All performance metrics reported in this study represent the mean values obtained across these ten folds and were further validated over multiple random seeds to ensure statistical stability.

The activation function used here maps the input values to the range [−1, 1], which is similar to the Sigmoid function. The output of the function is centered around 0, which helps reduce the learning rate and enhances learning speed. The output from the hidden layer is processed by the following activation function:

(3) $$\tanh\left(x\right)=\frac{e^{x}-e^{-x}}{e^{x}+e^{-x}}$$

The model uses Mean Squared Error (MSE) as the loss function. For the inference model of a single class, since the dataset is relatively small, the trainlm function in MATLAB 2024a is employed. This function uses the Levenberg-Marquardt algorithm, which is known for its fast convergence. The training parameters include a maximum iteration count (epochs) of 1000, a learning rate (η) of 0.005, and an error threshold (goal) of $10^{-16}$.

**Figure 4:**
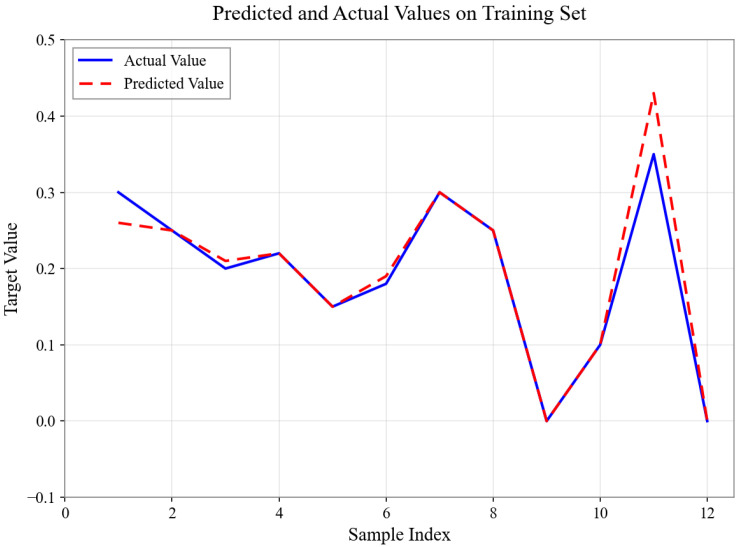
Predicted and Actual Values on Training Set with Category 24.

**Figure 5:**
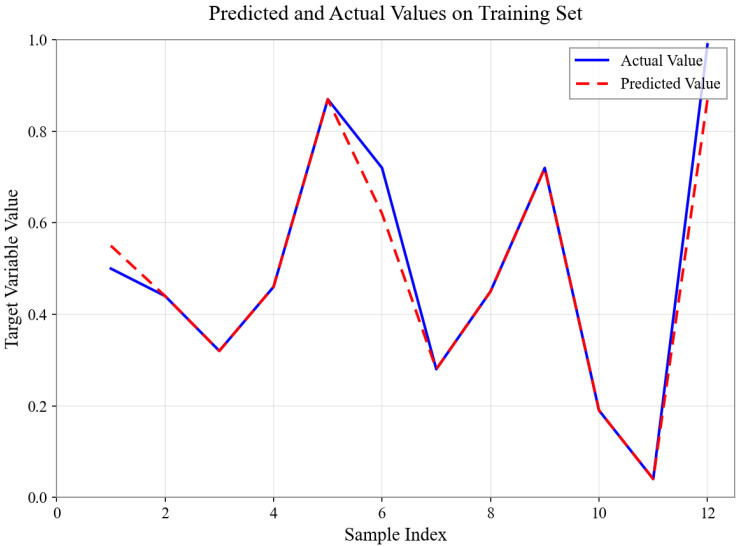
Predicted and Actual Values on Training Set with Category 41.

**Figure 6:**
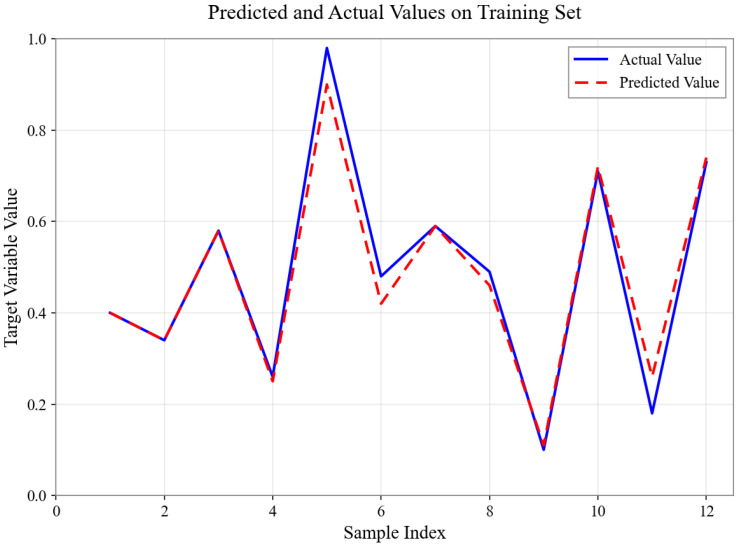
Predicted and Actual Values on Training Set with Category 52.

**Figure 7:**
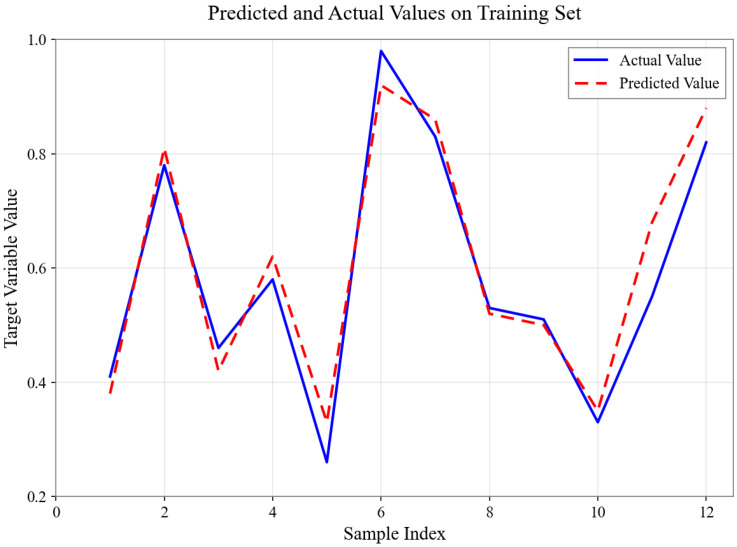
Predicted and Actual Values on Training Set with Category 67.

**Figure 8:**
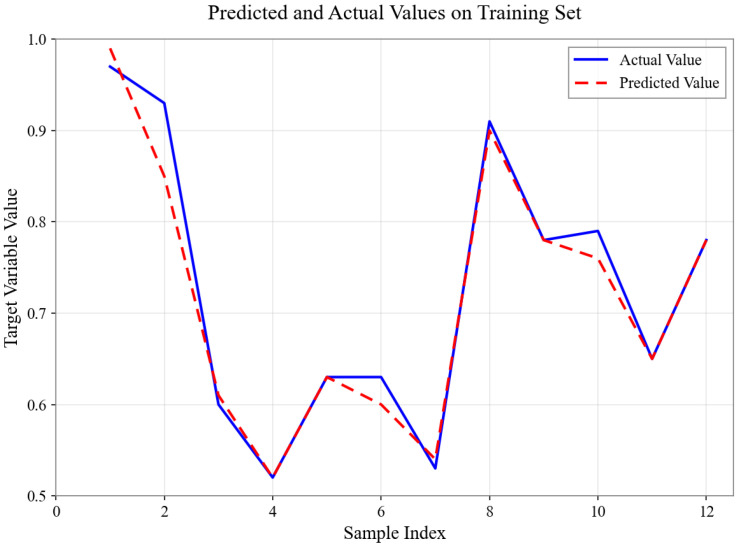
Predicted and Actual Values on Training Set with Category 79.

**Figure 9:**
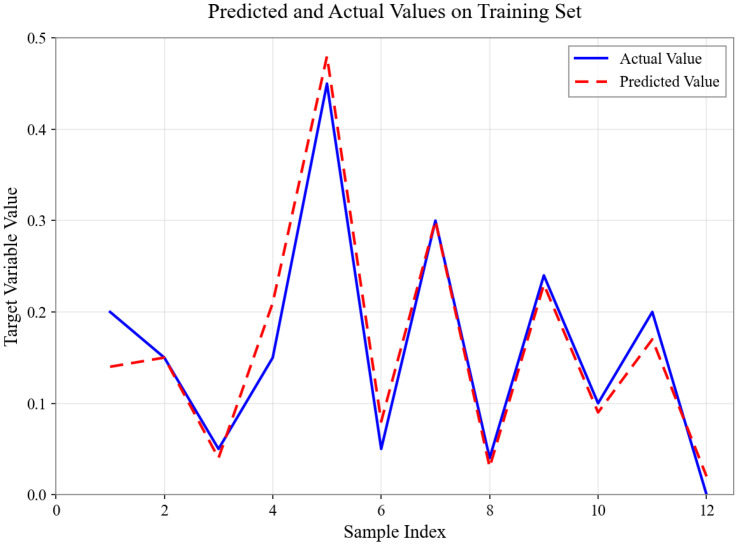
Predicted and Actual Values on Training Set with Category 188.

As shown in Figure 4, Figure 5, Figure 6, Figure 7, Figure 8 and Figure 9, for the BP neural network inference models constructed for categories 24, 41, 52, 67, 79, and 188, the Root Mean Square Error (RMSE) of the predicted outputs compared to the actual values is controlled between 0.00035 and 0.00073, while the coefficient of determination ($R^{2}$) remains within the high range of 0.9294 to 0.9745. The experimental results indicate that, thanks to the hierarchical architecture design of the Hybrid framework of SVM and BPNN, this machine learning-based inference model is capable of capturing the complex coupling relationships between environmental features and passability even when training data is scarce. It exhibits stronger adaptability when dealing with high-dimensional coupled features and dynamic environments.

This high level of accuracy is primarily attributed to the characteristics of the traversability mapping task, which resides in a relatively low-dimensional feature space with high data fidelity and low redundancy. Since the input environmental variables and the resulting traversability coefficient *K* follow consistent physical and computational patterns established in our framework, the neural network is capable of converging to a near-optimal approximation with minimal residual variance. Specifically, the relationship between the inputs *X* and the output *K* is largely deterministic, allowing the model to capture the underlying mapping with high fidelity, as reflected in the extremely low values of the residual sum of squares:

(4) $$RSS=\sum_{i=1}^{n}{\left(K_{i}-{\hat{K}}_{i}\right)}^{2}\approx 0$$

where $K_{i}$ and ${\hat{K}}_{i}$ denote the ground-truth and predicted traversability coefficients, respectively. These results confirm that the proposed learning module provides a reliable and precise mechanism for fusing multi-layer environmental information into a unified traversability index.

For complex wilderness environments where the road network is incomplete and the road network planning problem cannot be solved based on the complete road network, we propose a method for subdividing and constructing road network models. By using a direct graph and applying network road length differences as key elements, it enhances the communication of dynamic route planning and uses frequency adjustment for valid routes. The method combines the optimization sequence of the Dijkstra algorithm to effectively search the longest and optimal path.

The road network map is constructed by extracting the road network from the map, which needs to contain road nodes and edges. The node represents the start and end points of a road, and the edge represents the road itself.

A node is represented as a 3-dimensional array A=(lon,lat,ID). Here, lon and lat represent the longitude and latitude coordinates of the node, and ID is the unique identifier for the node. The ID is used to locate the node in the entire road network for correct identification. The edge between nodes is represented as a 2-dimensional array B=(u,v), where *u* and *v* represent two nodes that are adjacent to each other. The edge connects the two adjacent nodes and represents the road between them. In this study, we use the Haversine formula to calculate the distance between nodes based on the spherical geometry model:

$$L=2R\cdot \arcsin\left(\sqrt{\sin^{2}\left(\frac{\Delta \mathrm{lat}}{2}\right)+\cos\left({\mathrm{lat}}_{1}\right)\cdot \cos\left({\mathrm{lat}}_{2}\right)\cdot \sin^{2}\left(\frac{\Delta \mathrm{lon}}{2}\right)}\right),$$

where *R* is the Earth’s radius, $\Delta \mathrm{lat}={\mathrm{lat}}_{2}-{\mathrm{lat}}_{1}$, $\Delta \mathrm{lon}={\mathrm{lon}}_{2}-{\mathrm{lon}}_{1}$, ${\mathrm{lat}}_{1},{\mathrm{lat}}_{2}$ represent the latitude coordinates of the two nodes, and ${\mathrm{lon}}_{1},{\mathrm{lon}}_{2}$ represent the longitude coordinates of the two nodes. In continuous routes, the road network can be evaluated by various other factors, such as traffic conditions. These factors can be used to derive weights in the network’s edge, providing additional information for the planning process. The road network graph is represented by a set of nodes and edges, which allows for the application of algorithms like the Dijkstra algorithm to find the optimal path between nodes in the network.

For the path from a single node to a target node in the road network, when the distance between the node and the target is greater than a predefined threshold (e.g., dis>50km as set in this study), the path analysis process is carried out. The value of dis helps determine the road network and path efficiency, and further analysis can optimize the selection of roads based on the calculated values. Consider the set of candidate nodes $p=[n_{1},n_{2},\ldots ,n_{m}]$, where $n_{i}$ is a node in the road network, and each node is associated with its distance. To analyze the next node selection, we calculate the new node’s longitude and latitude using the following formula:

$${\mathrm{lon}}_{new}={\mathrm{lon}}_{i}+\frac{d}{L_{i+1}}\cdot \left({\mathrm{lon}}_{i+1}-{\mathrm{lon}}_{i}\right),$$

$${\mathrm{lat}}_{new}={\mathrm{lat}}_{i}+\frac{d}{L_{i+1}}\cdot \left({\mathrm{lat}}_{i+1}-{\mathrm{lat}}_{i}\right),$$

where *d* represents the distance from the current node to the next node, and $L_{i+1}$ is the distance between the current and next node. The network structure is based on node sets G=(V,E), where *V* is the set of nodes and *E* is the set of edges. The road network is represented as a graph structure, which provides a framework for the next step of network modeling.

**Figure 10:**
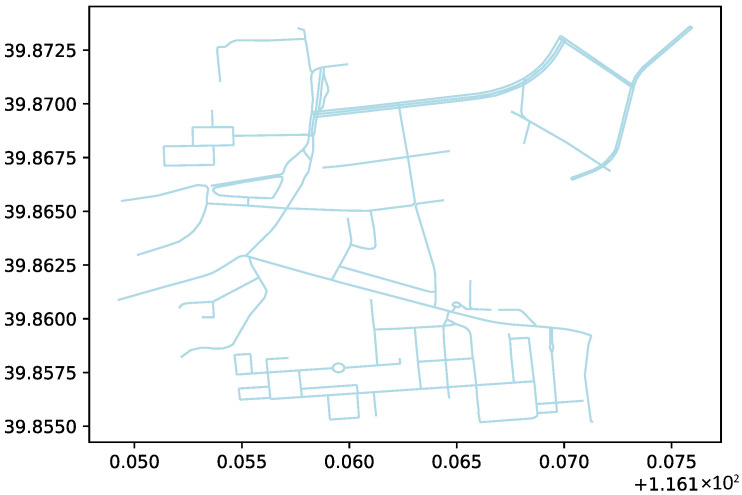
Basic road network used in this experiment.

**Figure 11:**
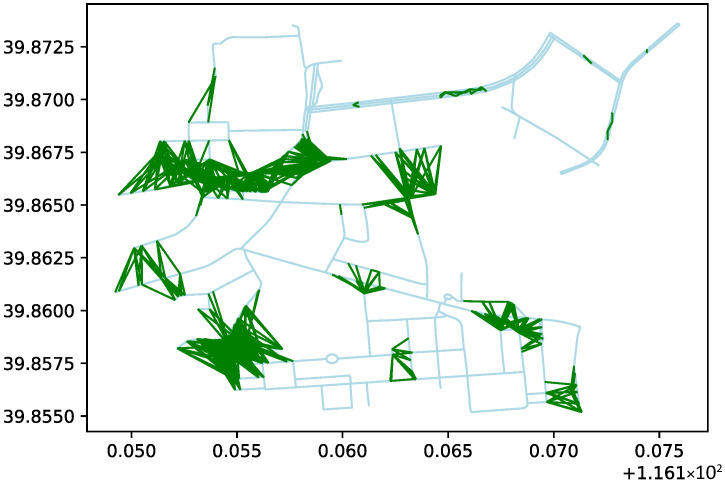
Expanded road network based on the method described in this paper.

In the road network constructed for the modeling base, starting from the node set in the road network, we assign weights based on the Euclidean distances between the nodes and their geographical locations. Consider the node set $B_{i}$ of the road network. For each node $B_{i}$, we calculate the corresponding Euclidean distance, $\mathrm{dis}(A-B_{i})$, between nodes, which provides the edge distance in the network. Using the set $B_{i}$, we can find the shortest path from the current node to the target node. This is achieved by using the Dijkstra algorithm, which calculates the shortest path between nodes in a network. The shortest path distance $L_{\mathrm{road}}$ is obtained using the formula:

$$L_{\mathrm{road}}=k\cdot \mathrm{dis}\left(A-B_{i}\right),$$

where *k* is a constant factor. In this case, the path length is determined by the weighted sum of distances along the edges. We continue this process until all paths between the nodes have been evaluated and the optimal path is selected. The basic road network used in this experiment is shown in Figure 10:

Based on the above foundation, the road network expansion was carried out using the method described in this paper, and the result is shown in Figure 11, where the green line represents the extended road line.

## 4. Convergence Analysis

Initially, a fundamental lemma is introduced, which is subsequently utilized to verify the convergence properties of the neural network with Gaussian function. Consider the objective function formulated as follows:

(5) $$\begin{array}{c} J\left(\Theta \right)=\frac{1}{2}\sum_{k=1}^{N}{\left(\tau_{k}-z_{k}\right)}^{2}=\sum_{k=1}^{N}L_{k}\left(\mathrm{fi}\cdot {\mathrm{fi}}_{k}\right), \end{array}$$

where $L_{k}$ denotes a function depending on $\tau_{k}=\mathrm{fi}\cdot {\mathrm{fi}}_{k}$, with $\mathrm{fi}\in R^{M}$ and ${\mathrm{fi}}_{k}\in R^{M}$. For the sake of brevity, the scalar product symbol “·” will be suppressed in the subsequent derivations. We designate the element-wise product operator “⊛” as:

$$\begin{array}{c} p⊛q=(p_{1}q_{1},p_{2}q_{2},\ldots ,p_{M}q_{M}), \end{array}$$

where $p=\left(p_{1},p_{2},\ldots ,p_{M}\right)\in R^{M}$ and $q=\left(q_{1},q_{2},\ldots ,q_{M}\right)\in R^{M}$.

The partial derivative with respect to the output weight vector fi is derived as:

(6) $$\begin{array}{c} \frac{\partial J(\Theta )}{\partial \mathrm{fi}}=\sum_{k=1}^{N}L_{k}^{\prime }\left({\mathrm{fifi}}_{k}\right){\mathrm{fi}}_{k}. \end{array}$$

Similarly, the gradient regarding the width parameter ${œ}_{m}$ is given by:

(7) $$\begin{array}{cc} \frac{\partial J(\Theta )}{\partial {œ}_{m}}= & -2\sum_{k=1}^{N}L_{k}^{\prime }\left(\mathrm{fi}{\mathrm{fi}}_{k}\right)\beta_{m}\\ \; & \beta_{m,k}\left(\left({Ø}_{k}-C_{m}\right)⊛\left({Ø}_{k}-C_{m}\right)⊛{œ}_{m}\right). \end{array}$$

For iteration steps t=0,1,2,⋯, sample indices 1≤k≤N, and dimensions 1≤m≤M, we define the following auxiliary variables:

(8) $$\begin{array}{c} A_{0}^{t,k}={\mathrm{fi}}^{t}\cdot {\mathrm{fi}}_{t,k},\;\Gamma^{t,k}={\mathrm{fi}}_{t+1,k}-{\mathrm{fi}}_{t,k},\\ {æ}_{m}^{k}={Ø}_{k}-C_{m},\;A_{m}^{t,k}={æ}_{m}^{k}⊛{œ}_{m}^{t}. \end{array}$$

**Lemma** **1.** *Assuming that the objective function is strongly convex and Lipschitz smooth, for any iteration step t=0,1,2,⋯, sample index 1≤k≤N, and neuron index 1≤m≤M, the following inequalities hold:*

(9) $$\begin{array}{cc} ∥A_{0}^{t,k}∥\leq \sqrt{M}K_{0}, & \;∥{æ}_{m}^{k}∥\leq K_{1}∥A_{m}^{t,k}∥\leq K_{1},\\ \; & ∥z_{k}∥\leq K_{1}, \end{array}$$

(10) $$\begin{array}{c} \sum_{k=1}^{N}L_{k}^{\prime }\left(A_{0}^{t,k}\right)\Delta {\mathrm{fi}}^{t}{\mathrm{fi}}_{t,k}=-\alpha {∥\frac{\partial J(\Theta^{t})}{\partial \mathrm{fi}}∥}^{2}, \end{array}$$

(11) $$\begin{array}{c} \begin{array}{c} \sum_{k=1}^{N}L_{k}^{\prime }\left(A_{0}^{t,k}\right)\Delta {\mathrm{fi}}^{t}\Gamma^{t,k}\leq K_{2}\alpha^{2}{∥\frac{\partial J\left(\Theta^{t}\right)}{\partial \Theta }∥}^{2}, \end{array} \end{array}$$

(12) $$\begin{array}{c} \sum_{k=1}^{N}L_{k}^{\prime }\left(A_{0}^{t,k}\right){\mathrm{fi}}^{t}\Gamma^{t,k}\leq \left(-\alpha +K_{3}\alpha^{2}\right)\sum_{m=1}^{M}{∥\frac{\partial J(\Theta^{t})}{\partial {œ}_{m}}∥}^{2}, \end{array}$$

(13) $$\begin{array}{c} \frac{1}{2}\sum_{k=1}^{N}L_{k}^{\prime \prime }\left(\zeta_{t,k}\right){\left(A_{0}^{t+1,k}-A_{0}^{t,k}\right)}^{2}\leq K_{4}\alpha^{2}{∥\frac{\partial J\left(\Theta^{t}\right)}{\partial \Theta }∥}^{2}, \end{array}$$

*where α represents the learning rate, $K_{r}\left(r=1,2,3,4\right)$ denote constants independent of t and k, and $\zeta_{t,k}$ is a scalar value lying between $A_{0}^{t,k}$ and $A_{0}^{t+1,k}$.*

**Proof.**  Based on the premise of Assumption (A1), the bounds are given by: $∥{\mathrm{fi}}^{t}∥\leq K_{0},∥C_{m}^{t}∥\leq K_{0},∥{œ}_{m}^{t}∥\leq K_{0}$, for all m=1,…,M and t=1,…

Let us introduce the following definitions:

(14) $$\begin{array}{cc} B_{\max}= & \max_{1\leq k\leq N}\left\{∥{Ø}_{k}∥,∥z_{k}∥\right\}, \end{array}$$

(15) $$\begin{array}{ll} \;\;\;\;\;\;\;K_{1}= & \max\left\{K_{0}+B_{\max},\left(K_{0}+B_{\max}\right)K_{0}\right\}. \end{array}$$

Utilizing these notations alongside Equation (8), the validity of the inequalities in (9) is readily apparent. □

Referring to Equation (6), the derivation of Equation (10) is straightforward:

$$\begin{array}{cc} \sum_{k=1}^{N}L_{k}^{\prime }\left(A_{0}^{t,k}\right)\Delta {\mathrm{fi}}^{t}{\mathrm{fi}}_{t,k} & =\frac{\partial J(\Theta^{t})}{\partial \mathrm{fi}}\cdot \left(-\alpha \frac{\partial J(\Theta^{t})}{\partial \mathrm{fi}}\right)\\ \; & =-\alpha {∥\frac{\partial J(\Theta^{t})}{\partial \mathrm{fi}}∥}^{2}. \end{array}$$

Subsequently, we proceed to verify inequality (11). From (5), (8) and (9), we deduce that:

(16) $$\begin{array}{c} L_{k}^{\prime }\left(z\right)=z-z_{k}, \end{array}$$

(17) $$\begin{array}{c} \left|L_{k}^{\prime }\left(A_{0}^{t,k}\right)\right|\leq \left(\sqrt{M}K_{0}+K_{1}\right). \end{array}$$

(18) $$\begin{array}{cc} \beta_{m,k}^{t}= & \exp\left(-\sum_{l=1}^{M}{\left(\chi_{k,l}-C_{m,l}\right)}^{2}{\left(\sigma_{m,l}^{t}\right)}^{2}\right)\\ = & \exp\left(-A_{m}^{t,k}\cdot A_{m}^{t,k}\right). \end{array}$$

Applying the Lagrange Mean Value Theorem yields:

$$\begin{array}{c} \begin{array}{cc} \Gamma_{m}^{t,k}= & {\mathrm{fi}}_{m,k}^{t+1}-{\mathrm{fi}}_{m,k}^{t}\\ = & \exp\left(-A_{m}^{t+1,k}\cdot A_{m}^{t+1,k}\right)-\exp\left(-A_{m}^{t,k}\cdot A_{m}^{t,k}\right)\\ = & \exp\left(\vartheta_{m}^{t,k}\right)\left(-\left(A_{m}^{t+1,k}\cdot A_{m}^{t+1,k}-A_{m}^{t,k}\cdot A_{m}^{t,k}\right)\right)\\ = & -\exp\left(\vartheta_{m}^{t,k}\right)\left(A_{m}^{t+1,k}+A_{m}^{t,k}\right)\cdot \left(A_{m}^{t+1,k}-A_{m}^{t,k}\right), \end{array} \end{array}$$

where $\vartheta_{m}^{t,k}$ lies in the interval between $-A_{m}^{t+1,k}\cdot A_{m}^{t+1,k}$ and $-A_{m}^{t,k}\cdot A_{m}^{t,k}$.

Consequently, we have:

(19) $$\begin{array}{c} \begin{array}{cc} \left|\Gamma_{m}^{t,k}\right|⩽ & 2K_{1}∥A_{m}^{t+1,k}-A_{m}^{t,k}∥\\ = & 2K_{1}∥{æ}_{m}^{k}⊛\left({œ}_{m}^{t+1}-{œ}_{m}^{t}\right)∥\\ ⩽ & 2K_{1}∥{æ}_{m}^{k}∥\cdot ∥{œ}_{m}^{t+1}-{œ}_{m}^{t}∥\\ ⩽ & 2K_{1}^{2}∥\Delta {œ}_{m}^{t}∥. \end{array} \end{array}$$

Thus, we can conclude:

(20) $$\begin{array}{cc} \; & \sum_{k=1}^{N}L_{k}^{\prime }\left(A_{0}^{t,k}\right)\Delta {\mathrm{fi}}^{t}\Gamma^{t,k}\\ \; & \leq \left(\sqrt{M}K_{0}+K_{1}\right)\sum_{k=1}^{N}∥\Delta {\mathrm{fi}}^{t,k}∥∥\Gamma^{t,k}∥\\ \; & \leq \frac{1}{2}\left(\sqrt{M}K_{0}+K_{1}\right)\sum_{k=1}^{N}\left({∥\Delta {\mathrm{fi}}^{t}∥}^{2}+{∥\Gamma^{t,k}∥}^{2}\right)\\ \; & \leq K_{2}\left({∥\Delta {\mathrm{fi}}^{t}∥}^{2}+\sum_{m=1}^{M}{∥\Delta {œ}_{m}^{t}∥}^{2}\right)\\ \; & =K_{2}\alpha^{2}{∥\frac{\partial J\left(\Theta^{t}\right)}{\partial \Theta }∥}^{2}, \end{array}$$

where the constant is defined as:

(21) $$\begin{array}{c} K_{2}=\frac{N}{2}\left(\sqrt{M}K_{0}+K_{1}\right)\max\left\{1,4K_{1}^{4}\right\}. \end{array}$$

This concludes the derivation of inequality (11). We now move to prove inequality (12).

Employing Taylor expansion results in:

(22) $$\begin{array}{cc} {\mathrm{fi}}^{t}\Gamma^{t,k}= & \sum_{m=1}^{M}\beta_{m}^{t}(\exp\left(-A_{m}^{t+1,k}\cdot A_{m}^{t+1,k}\right)\\ \; & -\exp(-A_{m}^{t,k}\cdot A_{m}^{t,k}))\\ \; & =\delta_{1}+\delta_{2}, \end{array}$$

with the terms defined as:

(23) $$\begin{array}{cc} \delta_{1}= & \sum_{m=1}^{M}\beta_{m}^{t}\beta_{m,k}^{t}\left(-\left(A_{m}^{t+1,k}\cdot A_{m}^{t+1,k}-A_{m}^{t,k}\cdot A_{m}^{t,k}\right)\right), \end{array}$$

(24) $$\begin{array}{cc} \delta_{2}= & \frac{1}{2}\sum_{m=1}^{M}\beta_{m}^{t}\exp\left({\tilde{\vartheta }}_{m}^{t,k}\right){\left(A_{m}^{t+1,k}\cdot A_{m}^{t+1,k}-A_{m}^{t,k}\cdot A_{m}^{t,k}\right)}^{2}, \end{array}$$

where ${\tilde{\vartheta }}_{m}^{t,k}$ is positioned between $-A_{m}^{t+1,k}\cdot A_{m}^{t+1,k}$ and $-A_{m}^{t,k}\cdot A_{m}^{t,k}$.

It is observed that:

(25) $$\begin{array}{cc} \; & A_{m}^{t+1,k}\cdot A_{m}^{t+1,k}-A_{m}^{t,k}\cdot A_{m}^{t,k}\\ \; & =\sum_{l=1}^{M}{\left(\chi_{k,l}-C_{m,l}\right)}^{2}\left(2\sigma_{m,l}^{t}+\Delta \sigma_{m,l}^{t}\right)\Delta \sigma_{m,l}^{t}\\ \; & =\sum_{l=1}^{M}{\left(\chi_{k,l}-C_{m,l}\right)}^{2}2\sigma_{m,l}^{t}\Delta \sigma_{m,l}^{t}\\ \; & \;+\sum_{l=1}^{M}{\left(\chi_{k,l}-C_{m,l}\right)}^{2}{\left(\Delta \sigma_{m,l}^{t}\right)}^{2} \end{array}$$

Using Equations (7), (8) and (25), we obtain:

$$\begin{array}{c} \begin{array}{cc} & \sum_{k=1}^{N}L_{k}^{\prime }\left(A_{0}^{t,k}\right)\delta_{1}\\ & =-2\sum_{k=1}^{N}L_{k}^{\prime }\left(A_{0}^{t,k}\right)\sum_{m=1}^{M}\beta_{m}^{t}\beta_{m,k}^{t}(\left({æ}_{m}^{k}⊛{æ}_{m}^{k}⊛{œ}_{m}^{t}\right)\\ & \cdot \Delta {œ}_{m}^{t})-\sum_{k=1}^{N}L_{k}^{\prime }\left(A_{0}^{t,k}\right)\sum_{m=1}^{M}\beta_{m}^{t}\beta_{m,k}^{t}{∥{æ}_{m}^{k}⊛\Delta {œ}_{m}^{t}∥}^{2}\\ & =\sum_{m=1}^{M}\frac{\partial J(\Theta )}{\partial {œ}_{m}}\cdot \Delta {œ}_{m}^{t}-\sum_{k=1}^{N}L_{k}^{\prime }\left(A_{0}^{t,k}\right)\sum_{m=1}^{M}\beta_{m}^{t}\beta_{m,k}^{t}{∥{æ}_{m}^{k}⊛\Delta {œ}_{m}^{t}∥}^{2} \end{array} \end{array}$$

Then, referencing (9) and (17), it holds that:

(26) $$\begin{array}{c} \begin{array}{c} \sum_{k=1}^{N}L_{k}^{\prime }\left(A_{0}^{t,k}\right)\delta_{1}\leq -\alpha \sum_{m=1}^{M}{∥\frac{\partial J(\Theta^{t})}{\partial {œ}_{m}}∥}^{2}+NK_{0}K_{1}^{2}\left(\sqrt{M}K_{0}+K_{1}\right)\sum_{m=1}^{M}{∥\Delta {{œ}_{m}}^{t}∥}^{2} \end{array} \end{array}$$

(27) $$\begin{array}{c} \begin{array}{cc} \sum_{k=1}^{N} & L_{k}^{\prime }\left(A_{0}^{t,k}\right)\delta_{2}\\ = & \frac{1}{2}\sum_{k=1}^{N}L_{k}^{\prime }\left(A_{0}^{t,k}\right)\sum_{m=1}^{M}\beta_{m}^{t}\exp\left({\tilde{\vartheta }}_{m}^{t,k}\right){\left(A_{m}^{t+1,k}\cdot A_{m}^{t+1,k}-A_{m}^{t,k}\cdot A_{m}^{t,k}\right)}^{2}\\ ⩽ & \frac{K_{0}\left(\sqrt{M}K_{0}+K_{1}\right)}{2}\sum_{k=1}^{N}\sum_{m=1}^{M}{\left(A_{m}^{t+1,k}\cdot A_{m}^{t+1,k}-A_{m}^{t,k}\cdot A_{m}^{t,k}\right)}^{2}\\ = & \frac{K_{0}\left(\sqrt{M}K_{0}+K_{1}\right)}{2}\sum_{k=1}^{N}\sum_{m=1}^{M}{\left[\left(A_{m}^{t+1,k}+A_{m}^{t,k}\right)\cdot \left(A_{m}^{t+1,k}-A_{m}^{t,k}\right)\right]}^{2}\\ ⩽ & 2K_{0}K_{1}^{2}\left(\sqrt{M}K_{0}+K_{1}\right)\sum_{k=1}^{N}\sum_{m=1}^{M}{∥{æ}_{m}^{k}⊛\Delta {œ}_{m}^{t}∥}^{2}\\ ⩽ & 2NK_{0}K_{1}^{4}\left(\sqrt{M}K_{0}+K_{1}\right)\sum_{m=1}^{M}{∥\Delta {œ}_{m}^{t}∥}^{2} \end{array} \end{array}$$

Combining the inequalities derived above allows us to formulate inequality (12) as follows:

(28) $$\begin{array}{cc} & \sum_{k=1}^{N}L_{k}^{\prime }\left(A_{0}^{t,k}\right){\mathrm{fi}}^{t}\Gamma^{t,k}\\ = & \sum_{k=1}^{N}L_{k}^{\prime }\left(A_{0}^{t,k}\right)\left(\delta_{1}+\delta_{2}\right)\\ \leq & -\alpha \sum_{m=1}^{M}{∥\frac{\partial J(\Theta^{t})}{\partial {œ}_{m}}∥}^{2}+NK_{0}K_{1}^{2}\left(\sqrt{M}K_{0}+K_{1}\right)\left(1+2K_{1}^{2}\right)\sum_{m=1}^{M}{∥\Delta {{œ}_{m}}^{t}∥}^{2}\\ \leq & \left(-\alpha +K_{3}\alpha^{2}\right)\sum_{m=1}^{M}{∥\frac{\partial J(\Theta^{t})}{\partial {œ}_{m}}∥}^{2}, \end{array}$$

where $K_{3}=NK_{0}K_{1}^{2}\left(\sqrt{M}K_{0}+K_{1}\right)\left(1+2K_{1}^{2}\right)$.

Note that $L_{k}^{\prime \prime }\left(z\right)=1$. Following a similar logic and utilizing Equation (19), the proof for inequality (13) proceeds as:

$$\begin{array}{c} \begin{array}{cc} & \frac{1}{2}\sum_{k=1}^{N}L_{k}^{\prime \prime }\left(\zeta_{t,k}\right){\left(A_{0}^{t+1,k}-A_{0}^{t,k}\right)}^{2}\\ = & \frac{1}{2}\sum_{k=1}^{N}{∥A_{0}^{t+1,k}-A_{0}^{t,k}∥}^{2}\\ = & \frac{1}{2}\sum_{k=1}^{N}{∥\Delta {\mathrm{fi}}^{t}\cdot {\mathrm{fi}}_{t+1,k}+{\mathrm{fi}}^{t}\cdot \left({\mathrm{fi}}_{t+1,k}-{\mathrm{fi}}_{t,k}\right)∥}^{2}\\ ⩽ & \sum_{k=1}^{N}\left({∥\Delta {\mathrm{fi}}^{t}\beta_{t+1,k}∥}^{2}+{∥{\mathrm{fi}}^{t}\Gamma^{t,k}∥}^{2}\right)\\ ⩽ & NM{∥\Delta {\mathrm{fi}}^{t}∥}^{2}+4NK_{0}^{2}K_{1}^{4}\sum_{m=1}^{M}{∥\Delta {œ}_{m}^{t}∥}^{2}\\ ⩽ & K_{4}\alpha^{2}{∥\frac{\partial J\left(\Theta^{t}\right)}{\partial \Theta }∥}^{2} \end{array} \end{array}$$

where $K_{4}=\max\left\{NM,4NK_{0}^{2}K_{1}^{4}\right\}$. The convergence is guaranteed if the step-size α satisfies the criteria in Equations (10)–(13). In practical scenarios, a learning rate of approximately 0.01 is utilized, which has been empirically verified to yield stable convergence. It should be noted that while the theory focuses on the global optimum of the regularized objective, the actual training process navigates the non-convex landscape to find a stable local minimum that represents a consistent traversability assessment. Failure to maintain these stability conditions could result in inaccurate *K* values, thereby compromising the safety of the generated path.

## 5. Numerical Experiments

**Figure 12:**
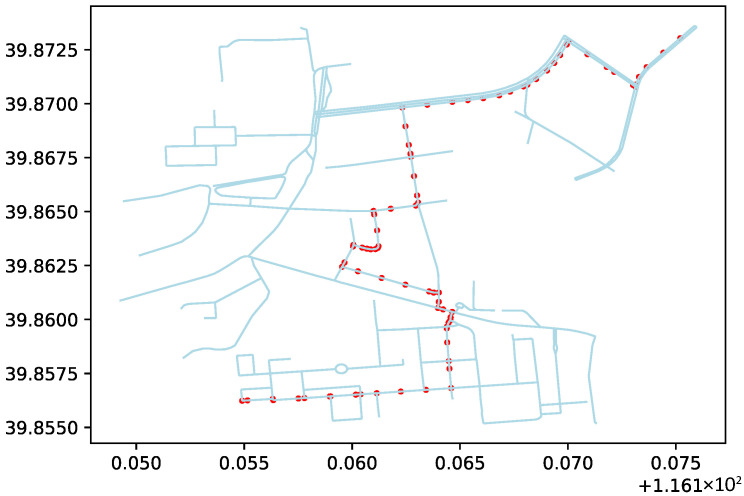
Original road network planning result.

In order to verify the effectiveness of the road network expansion algorithm, this study extracted the road network data of a certain urban-rural combined area and constructed the road network model in the experimental region. The road network has multiple dead ends, and the adjacent road network is sparse. The starting point is located in the lower left corner of the map, where the road planning algorithm is applied. The experimental setup is as follows: the road network distance dis=50m, the line segment length L=800m, the road length factor α=15, and the sampling number $n_{\mathrm{cont}}=400$. The road network after expansion was used in Dijkstra’s algorithm to find the optimal path. The result of the road network optimization is shown in Figure 12, where the red dots in the figure represent the key points of the planned path.

### 5.1. Global Path Planning Algorithm with or Without Road Networks

In the previous works, the path planning framework based on expanded road networks significantly improved network integrity through direct road connections and key node filtering. However, in complex wilderness environments characterized by typical scenarios such as “roads are present but impassable” and “no roads available”, relying solely on road network models still has limitations. By establishing virtual connections between road network nodes and tile center points, a hybrid model of ’hard road network + soft passability’ is formed. This model provides redundant options for specialized vehicles to avoid threats and traverse complex terrain.

In complex off-road environments where no road network is available, path planning relies on the analysis of terrain passability. This paper uses a hexagonal tile map to represent off-road environments without a road network. Compared to traditional square grid maps, hexagonal tile maps offer advantages such as higher coverage area, consistent adjacency relationships, compact structure, and better angular resolution, making them more suitable for research on vehicle passability and path planning under off-road conditions.

**Figure 13:**
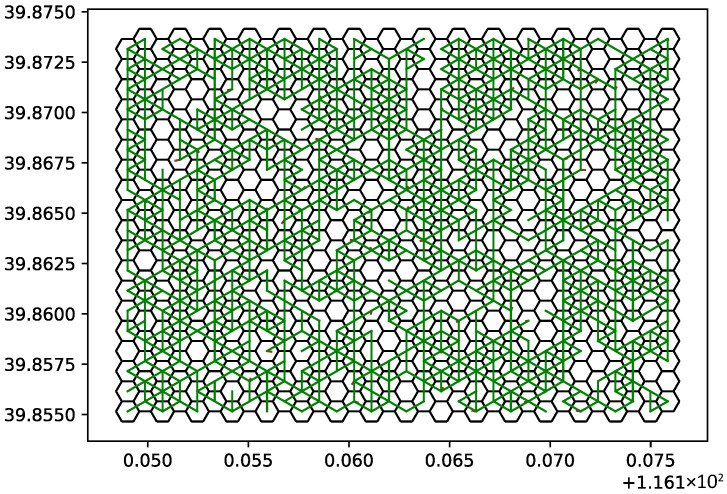
Randomly generated passable roads in the hexagonal tile map.

To express the passability of off-road areas, virtual connections are established between the center points of hexagonal tiles. These connections represent passable paths after evaluation. The generation of these paths is based on the construction rules of the hexagonal tile map, by connecting the center points of adjacent hexagons. In the simulation study, a circular area with a radius of 1050 m, centered at geographic coordinates 116.161394° E, 39.864316° N, was designated as the research area. In practical applications, the passability of each region on the hexagonal tile map should be evaluated based on actual terrain exploration results. To validate the algorithm logic, random numbers were introduced in this paper to randomly generate passable roads, as shown by the green lines in Figure 13.

### 5.2. Path Planning Algorithm for Road Network Integration with Off-Road Environments

In complex off-road environments, path planning for various terrain types often requires the integration of road network data with the terrain’s variability. To fully explore the off-road traversal capabilities of specialized vehicles, a method based on hybrid road network integration is proposed, where both conventional road network data and off-road environment features are integrated. This paper presents a method that combines hexagonal tile maps with traditional road network models to create a hybrid model for path planning in environments where there are no existing roads. In this method, virtual connections are created between the centers of hexagonal tiles, forming a “hard road network + soft passability” hybrid model. This model provides redundant options for specialized vehicles to bypass threats and traverse complex terrain.

In off-road environments without a road network, path planning depends on analyzing the terrain’s passability. This paper uses a hexagonal tile map to represent environments without a road network. Compared to traditional square grid maps, hexagonal tile maps offer several advantages, such as better coverage, consistent adjacency relationships, compact structure, and improved angular resolution, making them more suitable for off-road vehicle path planning.

**Figure 14:**
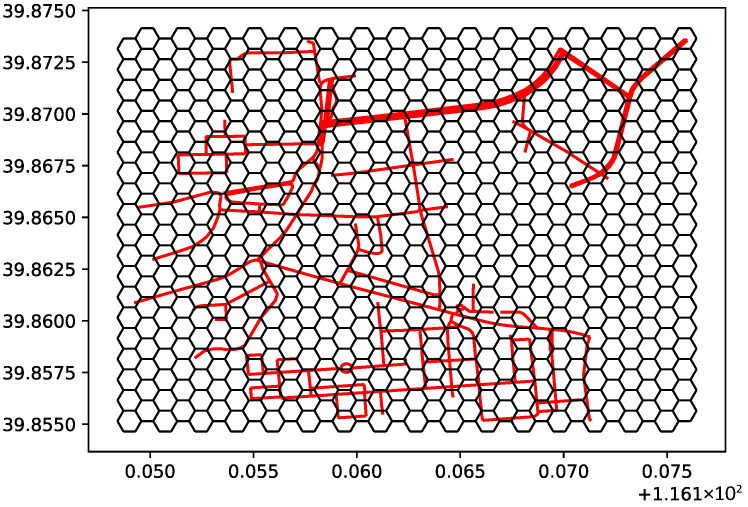
Road network integration with the terrain map using the random path generation method.

To represent the passability of the off-road area, virtual connections are established between the center points of the hexagonal tiles. These connections represent passable paths after evaluation. The paths are generated based on the rules for constructing the hexagonal tile map, connecting the centers of adjacent hexagons. In the simulation study, a circular area with a radius of 1050 m, centered at geographic coordinates 116.161394° E and 39.864316° N, was designated as the research area. In practical applications, the passability of each region in the hexagonal tile map should be evaluated based on actual terrain exploration results. To verify the algorithm’s logic, a random number generator was introduced to randomly generate passable paths, shown as red lines in Figure 14.

#### Simulation with Road Network Integration

**Figure 15:**
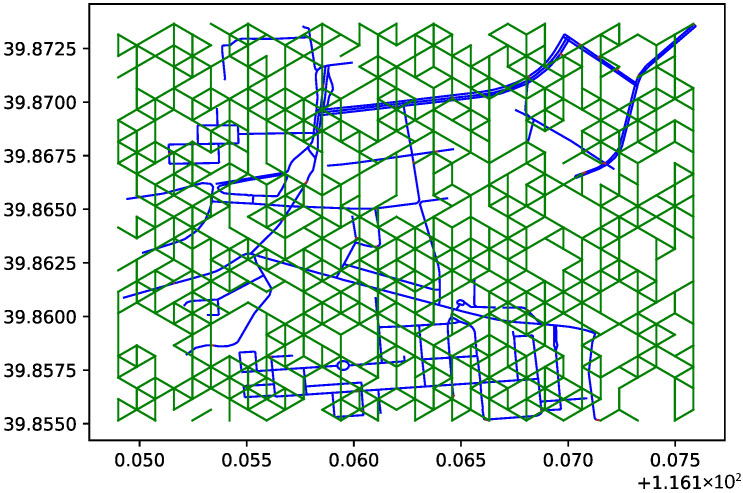
Road network integration result with random path generation.

**Figure 16:**
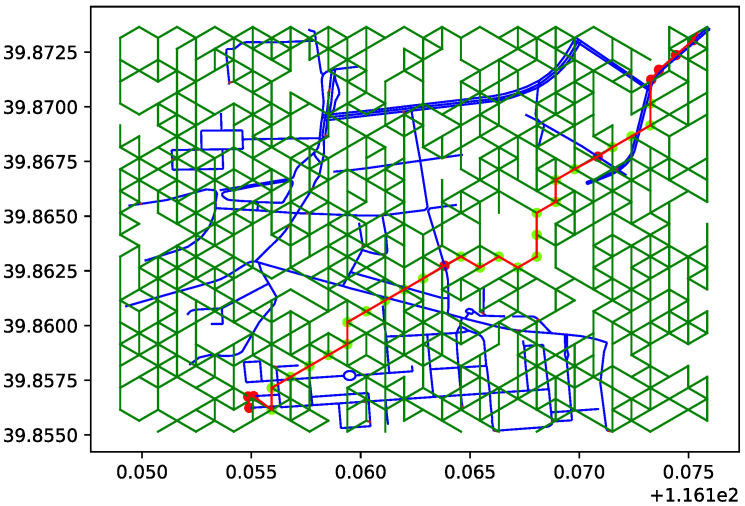
Dijkstra algorithm-based road network planning result.

By introducing a random number generator, random paths can be generated, as shown in the road network integration map in Figure 15 with blue lines:

The core idea of this algorithm is based on the connection of conditions, where the center of the hexagonal tile map is connected to the road network nodes. A “virtual road” is constructed, which forms a hybrid between the road network and the tile map. Using the Dijkstra algorithm for path planning, various types of off-road paths are identified and used in the expanded road network, providing options for vehicle movement. When constructing the expanded road network, the Dijkstra algorithm was used to identify the optimal path between nodes in the network, as shown in Figure 16, where the blue line represents the optimization process, and the red line represents the final optimal path.

In this case, the total road length of the path is 2895 m, with a deviation of 3070 m for the road network’s expansion. The final length of the road after expansion was 2530 m, which shows that the path expansion technique leads to a reduction in the total length of the road. After applying the Dijkstra algorithm to the path, the planning result optimized the path by selecting the shortest path based on the criteria. The optimization result is clearly better than the previous, as the road network used for calculation has more valid paths. At the same time, the method ensures that the path is navigable and adjusts for obstacles. This results in a more efficient route planning process, allowing vehicles to bypass complex terrain areas, significantly reducing the total travel time for the vehicle.

Through the simulation verification above, it is clear that the path planning algorithm based on road network integration provides advantages over single-road network planning. Road network integration offers flexibility in providing optimal off-road routes, but in practical applications, a fully connected road network may still present possible risks for vehicles. Therefore, even though the model using road network integration ensures passability, it is still influenced by environmental conditions, such as damaged roads or blocked paths. This results in an integrated model that is more adaptable to complex wilderness environment conditions. The results presented in Table 18 highlight the performance of different road network models based on their optimal path length. By comparing the proposed hybrid model against single-domain baselines (road-only and terrain-only), we demonstrate the significant gains achieved through multi-source information fusion. The algorithm achieves a practical runtime within seconds across all tested scenarios. While the current evaluation focuses on path length and information synergy, expanding the framework to include additional search-based baselines and optimizing for metrics such as feasibility and risk-averse costs remain key objectives for our future research.

In practical applications, the system can continuously collect environmental data, using real-time dynamic road network updates to adjust the vehicle’s path accordingly. This allows for optimal path planning under complex wilderness environment conditions by dynamically adapting to the changes in road network availability and terrain obstacles. The dynamic responsiveness of the proposed system is currently achieved through full recomputation of the path whenever the road network or environmental constraints are updated.

## 6. Conclusions

This paper has presented a hybrid global path planning framework tailored for specialized autonomous platforms operating in unstructured and post-disaster environments where infrastructure is often compromised. By establishing a fine-grained traversability evaluation model based on vehicle–terrain matching, the proposed method effectively quantifies the interaction between the platform’s kinematic constraints and complex topography, such as steep slopes and ravines. Furthermore, the novel integration of a road network expansion strategy with off-road search algorithms addresses the limitations of traditional planners, enabling a seamless transition between efficient on-road transit and flexible cross-country maneuvering to maximize the utility of high-mobility vehicles. In future work, we aim to enhance the system’s adaptability by incorporating real-time perception data to address dynamic environmental hazards. Additionally, we plan to validate the algorithm’s robustness through field experiments on physical platforms under authentic complex terrain conditions. For broader applications involving large-scale maps, the system’s scalability can be further enhanced by incorporating incremental update mechanisms and distributed parallel computing strategies, which are identified as key directions for our future work to optimize the computational budget and response frequency.

## Figures and Tables

**Table 1:** Correspondence between Traversability Coefficient and Traversability Capability.

Traversability Coefficient	Traversability Capability
0.8≤K≤1	Easily Passable
0.4≤K<0.8	Passable
0≤K<0.4	Impassable

**Table 15:** Classification of Terrain Feature Dataset.

Category	Vertical Obstacles	Trenches	Longitudinal Slope	Side Slope
1	1	1	rand	rand
2	rand	1	1	1
3	1	rand	1	1

**Table 16:** Classification of Surface Feature Datasets.

Category	Water Depth	Surf. Width	Struct. Road	Grassland	Shrubs	Snow & Ice	Dry Surf.	Water-Cont. Surf.
1	1	1	0.9	1	1	1	1	1
2	1	1	1	0.6	1	1	1	1
3	1	1	1	1	0.1	1	1	1
4	1	1	1	1	1	0.4	1	1
5	1	1	1	1	1	1	rand	1
6	1	1	1	1	1	1	1	rand
7	rand	rand	0.9	1	1	1	1	1
8	rand	rand	1	0.6	1	1	1	1
9	rand	rand	1	1	0.1	1	1	1
10	rand	rand	1	1	1	1	1	rand

**Table 17:** Category 24 Environmental Feature Data.

Altitude	Vert. Obs.	Trenches	Long. Slope	Side. Slope	Water Depth	Surf. Width	Struct. Road	
0.4	1	1	0.56	0.14	1	1	1	
0.35	1	1	0.3	0.96	1	1	1	
0.53	1	1	0.34	0.38	1	1	1	
0.31	1	1	0.58	0.91	1	1	1	
0.73	1	1	0.34	0.03	1	1	1	
0.81	1	1	0.29	0.37	1	1	1	
0.64	1	1	0.87	0.22	1	1	1	
0.36	1	1	0.39	0.81	1	1	1	
0.08	1	1	0.32	0.53	1	1	1	
0.17	1	1	0.35	0.05	1	1	1	
**Grass- Land**	**Shrubs**	**Snow & Ice**	**Dry Surf.**	**Water Cont. Surf.**	**Veg. Fact.**	**Mine Fact.**	**Fire Fact.**	**Comp. Value**
1	1	0.4	1	1	1	1	1	0.3
1	1	0.4	1	1	1	1	1	0.25
1	1	0.4	1	1	1	1	1	0.2
1	1	0.4	1	1	1	1	1	0.22
1	1	0.4	1	1	1	1	1	0.15
1	1	0.4	1	1	1	1	1	0.18
1	1	0.4	1	1	1	1	1	0.3
1	1	0.4	1	1	1	1	1	0.25
1	1	0.4	1	1	1	1	1	0
1	1	0.4	1	1	1	1	1	0.1

**Table 18:** Comparison of Optimal Path Lengths for Different Road Network Models.

Road Network Model	Path Length (m)
Road Network Model	4333
Road Network Expansion	3560
Hexagonal Off-Road Map	2530
Integrated Road Network Model	2895

## Data Availability

Data is contained within the article.
